# The Predictive Value of TyG-BMI and TG/HDL-C for Metabolic Dysfunction-Associated Steatotic Liver Disease in Obstructive Sleep Apnea: A Single-Center Retrospective Cohort Analysis

**DOI:** 10.3390/jcm15051859

**Published:** 2026-02-28

**Authors:** Furong Lv, Tong Li, Fei Zou, Xiuli Chen, Haiying Tang, Jingwei Mao

**Affiliations:** 1 Department of Gastroenterology, First Affiliated Hospital of Dalian Medical University, Dalian 116011, China; lvfr0621@163.com (F.L.); litong2024@dmu.edu.cn (T.L.); chenxl@dmu.edu.cn (X.C.); 2 Infectious Diseases Department, Dalian Public Health Clinical Center, Dalian 116038, China; 3 Department of Respiratory and Critical Disease, Respiratory Sleep Disorder Center, First Affiliated Hospital of Dalian Medical University, Dalian 116011, China; zoufei@dmu.edu.cn

**Keywords:** obstructive sleep apnea, metabolic dysfunction-associated steatotic liver disease, insulin resistance, triglyceride-glucose index with body mass index, triglyceride to high-density-lipoprotein-cholesterol ratio

## Abstract

**Background/Objectives**: This study aimed to evaluate the predictive value of the triglyceride-glucose index with body mass index (TyG-BMI) and the triglyceride-to-high-density lipoprotein-cholesterol (TG/HDL-C) ratio for predicting the occurrence of metabolic dysfunction-associated steatotic liver disease (MASLD) in obstructive sleep apnea (OSA). **Methods**: Data from patients diagnosed with OSA were analyzed in this retrospective cohort study. The participants were stratified into two groups: OSA alone and OSA with MASLD. The clinical characteristics and polysomnography data were collected. TyG-BMI and TG/HDL-C ratios were categorized into tertiles. Logistic regression and receiver operating characteristic (ROC) curve analyses were conducted to identify risk factors and assess their predictive performance for MASLD in OSA. **Results**: Among the 133 patients with OSA, 104 (78.2%) were diagnosed with MASLD. Multivariate analysis identified alanine aminotransferase (ALT), alkaline phosphatase, and TyG-BMI as independent risk factors for MASLD development in patients with OSA. Both TyG-BMI and TG/HDL-C ratio were significant predictors of MASLD in this patient population. The optimal cut-off values for TyG-BMI and TG/HDL-C ratio were 0.546 (sensitivity, 79.6%; specificity, 75.0%) and 0.539 (sensitivity, 93.2%; specificity, 60.7%), respectively. Combining TyG-BMI with ALT improved the predictive accuracy, yielding a cutoff of 0.696 (sensitivity, 76.7%; specificity, 92.9%). Similarly, the combination of TG/HDL-C ratio with ALT resulted in a cutoff value of 0.728 (sensitivity, 83.5%; specificity, 89.3%). **Conclusions**: TyG-BMI and the TG/HDL-C ratio are effective predictors of MASLD in patients with OSA. A combined model incorporating these indices with ALT levels demonstrated enhanced predictive accuracy for MASLD in this population. These indices are well-suited for risk stratification in resource-constrained settings facing a rising dual burden of OSA and MASLD.

## 1. Introduction

Obstructive sleep apnea (OSA) is a respiratory disorder characterized by repeated episodes of pharyngeal collapse during sleep, leading to chronic intermittent hypoxemia (CIH), which increases sympathetic activity and sleep fragmentation [1]. Consequently, OSA increases the risk of cardiovascular comorbidities and other systemic disorders [2]. A wide variety of potential mechanisms related to OSA that may contribute to co-morbidity have been studied, ranging from pathophysiological features of OSA, including CIH, fluctuations in intrathoracic pressure, and recurring arousals, to cell and molecular mechanisms that include sympathetic excitation, systemic inflammation, insulin resistance (IR), and oxidative stress, in addition to metabolic and endothelial dysfunction [3]. There is growing evidence from clinical studies that OSA is associated with the prevalent and incident components of metabolic syndrome and the metabolic syndrome itself [4].

In 2023, after four rounds of Delphi surveys, the term metabolic dysfunction-associated steatotic liver disease (MASLD) was chosen to replace non-alcoholic fatty liver disease (NAFLD) [5]. An international expert consensus subsequently supported updating the existing NAFLD code (DB92) in the ICD-11 to reflect this change [6]. MASLD is diagnosed in the presence of hepatic steatosis accompanied by metabolic risk factors, including obesity, IR, hypertension, and hypertriglyceridemia. These metabolic drivers are central to the pathogenesis of MASLD and contribute to its growing global burden. MASLD, currently the most prevalent chronic liver disease worldwide, is associated with a markedly increased risk of both liver-related mortality and hepatocellular carcinoma (HCC)-specific mortality [7]. At the cellular level, hepatic lipid accumulation induces lipotoxicity, which activates oxidative stress and pro-inflammatory signaling pathways that propagate intercellularly, ultimately driving progression from simple steatosis to inflammation and fibrosis [8].

The clinical significance of MASLD extends beyond the liver. Studies have demonstrated that NAFLD (now termed MASLD) is associated with more severe outcomes in other conditions, such as acute ischemic stroke, with its presence linked to greater neurologic severity and poorer functional recovery [9]. This systemic impact underscores the importance of early identification and intervention in at-risk populations. Recognition of the association between OSA and liver disease is not new. In 2012, a landmark review synthesized the proposed mechanisms linking OSA to NAFLD, advancing the hypothesis that the liver is a critical end-organ of OSA-induced injury [10]. In recent years, investigations into this relationship have continued, and recent research has confirmed that OSA is linked to the onset and progression of MASLD, independent of obesity or other shared risk factors [11,12]. Despite these advances, significant knowledge gaps remain, including an incomplete mechanistic understanding of the causal pathways, the lack of robust biomarkers for risk stratification in patients with OSA, and the need to reassess these associations within the contemporary MASLD framework.

In China, the rising prevalence of both OSA and MASLD represents an urgent public health challenge [13,14], with IR serving as a key pathophysiological link between these conditions. OSA-induced tissue hypoxia promotes IR, which in turn accelerates hepatic lipid accumulation and downstream cardiometabolic dysregulation. A recent Global Burden of Disease Study reported that population-level exposure to major metabolic risk factors—particularly elevated body mass index and fasting plasma glucose—has increased substantially across many regions over the past three decades, underscoring the need for scalable preventive strategies [15]. In this context, simple and reliable surrogate markers of IR, such as the triglyceride-glucose index combined with body mass index (TyG-BMI) and the triglyceride-to-high-density lipoprotein cholesterol (TG/HDL-C) ratio, have emerged as promising tools for risk stratification [16]. TyG-BMI and TG/HDL-C ratio have significant predictive value for the early screening and diagnosis of MASLD [17,18]. However, research on their application in OSA is scarce. This study aimed to assess the predictive value of TyG-BMI and TG/HDL-C ratio in patients with OSA and MASLD, thereby providing a basis for the early identification of potential MASLD within the OSA population and facilitating timely intervention.

## 2. Materials and Methods

### 2.1. Study Setting and Patient Enrollment

**Figure 1:**
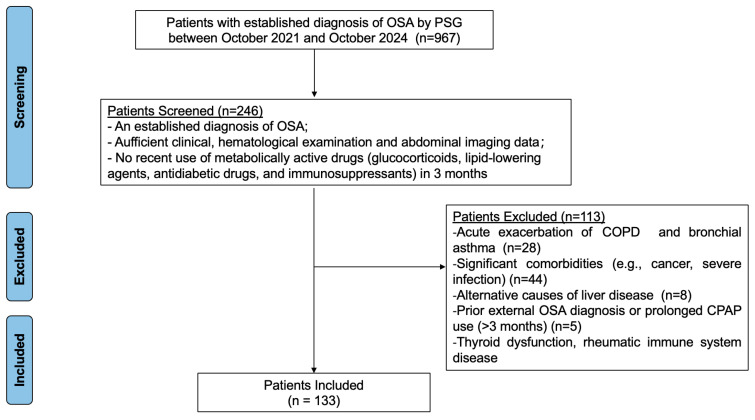
Study flowchart. Note: OSA, obstructive sleep apnea; PSG, polysomnography; COPD, chronic obstructive pulmonary disease; CPAP, continuous positive airway pressure.

We conducted a retrospective observational cohort study at a single center. The study population consisted of adults who underwent polysomnography at the Respiratory Sleep Disorder Center of the First Affiliated Hospital of Dalian Medical University (Dalian, China) between October 2021 and October 2024 and were initially diagnosed with OSA as defined by standard clinical guidelines [19]. The diagnosis of MASLD was established using the European Association for the Study of the Liver, European Association for the Study of Diabetes, and European Association for the Study of Obesity criteria [20]. Patients were excluded for any of the following criteria: prior external OSA diagnosis or prolonged continuous positive airway pressure use (>3 months); acute exacerbation of chronic respiratory conditions including chronic obstructive pulmonary disease or bronchial asthma; excessive alcohol intake (≥30 g/d for men and ≥20 g/d for women); alternative causes of liver disease; significant comorbidities (e.g., cancer, severe infection); recent use of metabolically active drugs (e.g., glucocorticoids, lipid-lowering agents, antidiabetic drugs, and immunosuppressants); or insufficient clinical data (Figure 1).

The reporting of our study conformed to the STROBE guidelines and the details of all patients were de-identified to ensure confidentiality. This study was approved by the Ethics Committee of the First Affiliated Hospital of Dalian Medical University (approval number: PJ-KS-KY-2025-40, 20 January 2025), and the requirement for informed consent was waived for this retrospective study.

### 2.2. Data Collection

Clinical data were retrospectively extracted from institutional electronic medical records (Yidu Cloud System and Huitai Report Data Center Platform). The demographic variables included age, sex, weight, height, medical history, and current medication use. Laboratory parameters assessed within 3 months before polysomnography included fasting plasma glucose (FPG), total cholesterol, TG, HDL-C, low-density lipoprotein cholesterol (LDL-C), alanine aminotransferase (ALT), aspartate aminotransferase (AST), alkaline phosphatase (ALP), and gamma-glutamyltransferase (GGT) levels. Abdominal ultrasonography and computed tomography scans were performed during this period.

### 2.3. Polysomnographic Monitoring and Calculated Indices

Sleep architecture was assessed using overnight polysomnography, from which parameters including the apnea–hypopnea index (AHI), oxygen saturation indices (MinSpO_2_, MeanSpO_2_, T90%), arousal index (ArI), and oxygen desaturation index (ODI) were obtained. We also calculated the following indices: BMI, defined as weight/height^2^; TyG index, calculated as (ln[TG(mg/dL) × FPG(mg/dL)]/2); TyG-BMI, calculated as TyG × BMI; and the TG/HDL-C ratio, defined as TG (mmol/L) divided by HDL-C (mmol/L).

### 2.4. Statistical Analysis

Statistical analyses were performed using SPSS software (version 27.0; IBM Corp., Armonk, NY, USA). Graphs were generated using GraphPad Prism (version 10.5.0; GraphPad Software, Boston, MA, USA). The normality of continuous variables was assessed using the Shapiro–Wilk test. Normally distributed data are presented as mean ± standard deviation and were compared using independent samples *t*-tests. Non-normally distributed data were summarized as medians with interquartile ranges [M (P25, P75)] and were compared using the Kruskal–Wallis H test. Categorical variables, such as sex, are described as frequencies and percentages (%) and were compared using the χ^2^ test. To identify factors associated with MASLD in patients with OSA, variables with a *p*-value < 0.05 in initial screenings were included in correlation analyses (Pearson or Spearman, as appropriate). Variables exhibiting collinearity (Variance Inflation Factor ≥ 10) were excluded. Univariate and multivariate logistic regression analyses were conducted to identify independent risk factors for MASLD in patients with OSA, with the results expressed as odds ratios (ORs) and 95% confidence intervals (CIs). Receiver operating characteristic curves were plotted to evaluate the predictive performances of TyG-BMI, TG/HDL-C ratio, and their combinations. The area under the curve (AUC) was calculated, and the optimal cutoff value was determined using the Youden index. Statistical significance was defined as a two-sided *p*-value of <0.05.

## 3. Results

### 3.1. Incidence of MASLD in Patients with OSA

**Figure 2:**
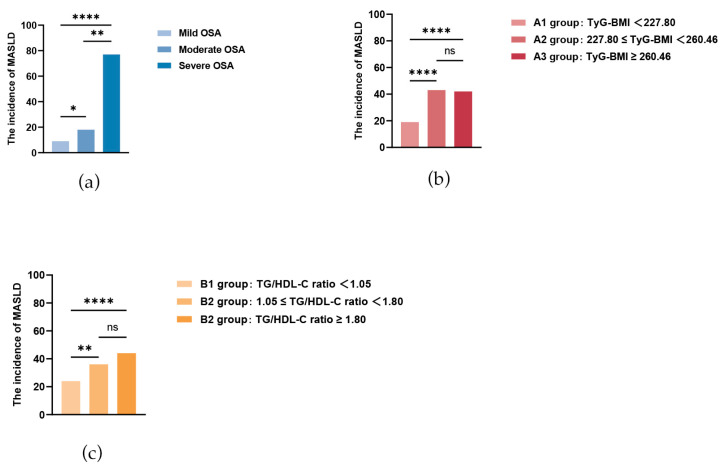
Assessment of MASLD incidence in OSA and risk stratification by TyG-BMI and TG/HDL-C ratio. (**a**) The incidence of MASLD in OSA; (**b**) Incidence of MASLD in different groups categorized by TyG-BMI; (**c**) Incidence of MASLD in different groups categorized by the TG/HDL-C ratio. *, *p* < 0.05; **, *p* < 0.01; ****, *p* < 0.00; ns, non-significant.

After rigorous screening, 133 eligible patients diagnosed with OSA were included in the study. OSA severity was classified per guideline standards as mild (AHI 5–15/h), moderate (AHI 15–30/h), or severe (AHI > 30/h). The cohorts included 24, 25, and 84 patients with mild, moderate, and severe OSA, respectively. Among them, 104 (78.2%) had concomitant MASLD (the OSA-with-MASLD group), whereas the remaining 29 (21.8%) constituted the OSA-only group. The prevalence of MASLD exhibited a significant stepwise increase across the severity grades (8.70%, 17.30%, and 74.00%, *p* < 0.001; Figure 2a).

### 3.2. Comparison of OSA Patients with and Without MASLD

Significant differences in the clinical characteristics were observed between the OSA-only and OSA-with-MASLD groups (Table 1). Compared to the OSA-only group, patients with OSA and MASLD were significantly younger, predominantly male, and had a higher average BMI. Their polysomnography parameters also indicated greater disease severity, with significantly elevated AHI, ArI, and ODI scores; prolonged T90%; and lower MinSpO_2_. Notably, this group also demonstrated a more adverse metabolic profile, including higher levels of ALT, AST, ALP, GGT, FPG, total cholesterol, TG, and LDL-C and lower HDL-C. Furthermore, IR indices were significantly higher in the OSA-with-MASLD group than in the OSA-only group. The median TyG-BMI was 252.61 (interquartile range [IQR] 231.93–280.46) versus 194.96 (IQR 169.45–229.65), and the median TG/HDL-C ratio was 1.58 (IQR 1.11–2.23) versus 0.64 (IQR 0.45–1.19) (all *p* < 0.001).

### 3.3. Comparison of Clinical Characteristics Across TyG-BMI and TG/HDL-C Ratio Tertiles

Participants were stratified into groups by either TyG-BMI (A1: <227.80; A2: 227.80–260.46; A3: ≥260.46) or the TG/HDL-C ratio (B1: <1.05; B2: 1.05–1.80; B3: ≥1.80), revealing a similar dose–response relationship with the MASLD incidence. In the TyG-BMI tertiles, the incidences of MASLD were 18.27%, 41.35%, and 40.39% in groups A1, A2, and A3, respectively. Similarly, across the TG/HDL-C ratio tertiles, MASLD incidence was 23.01%, 34.62%, and 42.3% in groups B1, B2, and B3, respectively. Post hoc analyses for both indicators confirmed that the incidence of MASLD was significantly higher in the middle and high tertiles than in the lowest tertile (all *p* < 0.001, Figure 2b,c), but no significant difference was observed between the middle and high tertiles (TyG-BMI: *p* = 0.431; TG/HDL-C: *p* = 0.175).

As summarized in Table 2, both TyG-BMI and TG/HDL-C ratio tertile groupings revealed broadly consistent patterns of significant differences across multiple metabolic and sleep-related parameters, including age, sex, BMI, AHI, ArI, ODI, MinSpO_2_, T90%, ALT, AST, GGT, TG, and HDL-C (all *p* < 0.05). Specifically, the highest tertile groups (A3 for TyG-BMI and B3 for TG/HDL-C) were consistently associated with younger age and higher BMI than the lowest tertiles. However, while LDL-C levels differed significantly across the TG/HDL-C ratio tertiles (*p* < 0.05), they did not vary significantly among the TyG-BMI groups. Similarly, MeanSpO_2_ showed significant differences across TyG-BMI tertiles but not across TG/HDL-C ratio groups. In contrast, ALP, FPG, and total cholesterol levels showed no significant differences across the tertiles in either grouping system (*p* > 0.05).

### 3.4. Risk Factors for OSA Combined with MASLD

**Figure 3:**
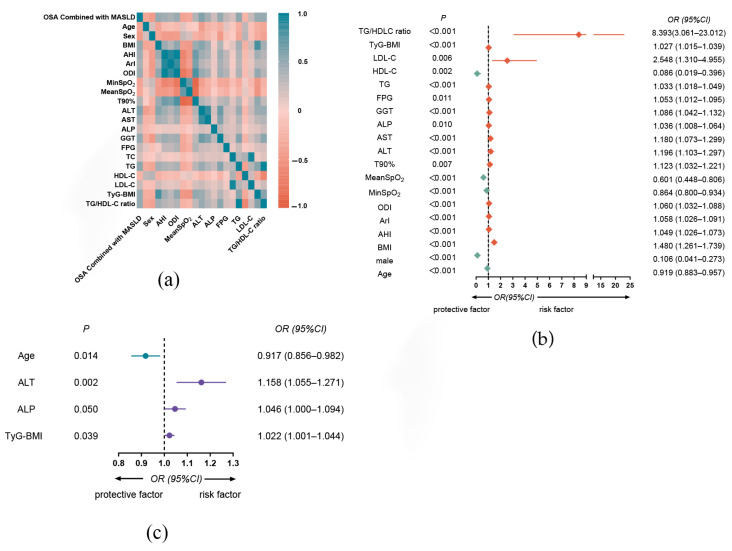
Risk factors and regression analyses for MASLD in patients with OSA. (**a**) Risk Factors for OSA Combined with MASLD; (**b**) The univariate binary logistic regression analysis of clinical data associated with incidence of MASLD in OSA; (**c**) The multivariate binary logistic regression of clinical data associated with incidence of MASLD in OSA.

Spearman correlation analysis was performed to identify factors associated with the presence of OSA combined with MASLD. As shown in Figure 3a, negative correlations were observed for age, male sex, MinSpO_2_, MeanSpO_2_, and HDL-C, with male sex demonstrating a moderate inverse correlation (r = −0.442) and the others showing weak correlations. In contrast, multiple variables showed positive correlations, among which BMI, AHI, ODI, ALT, AST, GGT, TG, TyG-BMI, and the TG/HDL-C ratio exhibited moderate correlations (0.4 < r < 0.60), while ArI, T90%, ALP, FPG, and LDL-C showed weak correlations (0.20 < r < 0.40).

Univariate logistic regression analysis indicated that all the aforementioned variables were significantly associated with the incidence of MASLD in patients with OSA (*p* < 0.05). Elevated TyG-BMI (OR 1.027, 95% CI, 1.015–1.039; *p* < 0.001) and TG/HDL-C ratio (OR 8.393, 95% CI, 3.061–23.012; *p* < 0.001) were significant risk factors for MASLD in patients with OSA (Figure 3b). To address multicollinearity, a diagnostic check was performed, leading to the exclusion of AHI and ODI owing to high Variance Inflation Factors (13.678 and 13.021, respectively; Supplementary Table S1). Subsequently, a multivariate binary logistic regression model was constructed by backward elimination. The dependent variables were the presence of OSA and the MASLD scores. The independent variables included age, sex, ArI, MinSpO_2_, MeanSpO_2_, T90%, ALT, AST, ALP, GGT, LDL-C, TyG-BMI, and TG/HDL-C ratio. The final model identified ALT (OR = 1.158, 95% CI: 1.055–1.271, *p* = 0.002), ALP (OR = 1.046, 95% CI: 1.000–1.094, *p* = 0.050), and TyG-BMI (OR = 1.022, 95% CI: 1.001–1.044, *p* = 0.039) as independent risk factors. In contrast, age was found to be a protective factor (OR = 0.917, 95% CI: 0.856–0.982, *p* = 0.014, see Figure 3c).

### 3.5. Predictive Value of TyG-BMI and Combined Factors for MASLD in OSA Patients

**Figure 4:**
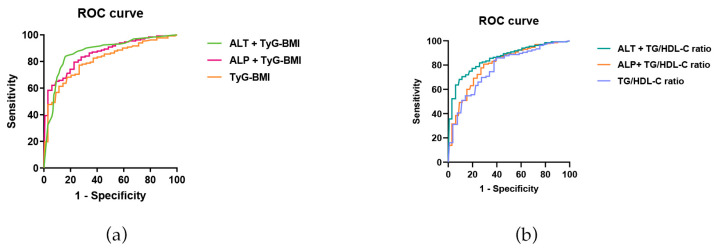
Comparison of ROC curves for different predictive models of MASLD. (**a**) ROC curves of TyG-BMI, TyG-BMI and ALT, and TyG-BMI and ALP fitting models. (**b**) ROC curves of TG/HDL-C ratio, TG/HDL-C ratio with ALT, and TG/HDL-C ratio.

The receiver operating characteristic curves generated using TyG-BMI, TyG-BMI and ALT, and TyG-BMI and ALP fitting models demonstrated AUC values of 0.826, 0.921, and 0.854, respectively (Table 3). These findings indicate that all three models can serve as predictive indicators of MASLD in individuals with OSA. Using the Youden index, the optimal cut-off value for the TyG-BMI model was determined to be 0.546, yielding a sensitivity of 79.6% and specificity of 75.0%. For the TyG-BMI and ALT fitting models, the optimal cutoff value was 0.696, with a sensitivity of 76.7% and specificity of 92.9%. The TyG-BMI and ALP fitting models had an optimal cutoff value of 0.595, with a sensitivity of 73.80% and specificity of 85.70%. Notably, the TyG-BMI and ALT fitting models exhibited the highest predictive accuracy and diagnostic value among the evaluated models (Figure 4a).

### 3.6. Predictive Value of TG/HDL-C Ratio and Combined Factors for MASLD in OSA Patients

The receiver operating characteristic curves constructed for the TG/HDL-C ratio, the TG/HDL-C ratio combined with the ALT fitting model, and the TG/HDL-C ratio combined with the ALP fitting model showed AUC values of 0.824, 0.920, and 0.840, respectively. These findings indicated that all three models were effective predictive indicators of MASLD in individuals with OSA. According to the Youden index, the optimal cut-off value for the TG/HDL-C ratio was determined to be 0.539, achieving a sensitivity of 93.20% and specificity of 60.70%. The optimal cutoff for the TG/HDL-C ratio and ALT fitting model was 0.728, with a sensitivity of 83.50% and a specificity of 89.30%. Similarly, the TG/HDL-C ratio and ALP fitting model had an optimal cut-off value of 0.562, with a sensitivity of 88.30% and a specificity of 67.90% (Table 3). Among these models, the TG/HDL-C ratio combined with ALT fitting model exhibited superior predictive accuracy for MASLD in individuals with OSA, offering higher specificity and diagnostic significance (Figure 4b).

## 4. Discussion

The recent shift in nomenclature from NAFLD to MASLD, with an emphasis on affirmative metabolic criteria rather than exclusion, has important implications for OSA research. While a body of evidence has accumulated over the past decade exploring the relationship between OSA and NAFLD, the transition to the definition of MASLD necessitates a re-evaluation of these associations under the new metabolic paradigm. The present study addresses this gap by providing contemporary evidence of the predictive value of metabolic indices for MASLD in patients with OSA.

In our study, patients with OSA and MASLD were predominantly men and had a higher BMI than patients with OSA only. Previous studies have established that sex and obesity are critical risk factors for OSA and MASLD [21,22,23]. Importantly, there was a notable correlation between OSA severity and MASLD, with a high comorbidity rate observed, especially among adults with obesity [24]. The prevalence of MASLD in patients with OSA is higher than that in the general population [25], with a significantly higher prevalence in men than in women [26]. This sex disparity may be attributed to the protective effects of estrogen in women. Patients in the OSA-with-MASLD group exhibited more pronounced sleep disturbances than those in the OSA-only group. Specifically, the OSA with MASLD group demonstrated elevated AHI levels, increased T90% during nighttime sleep, reduced MinSpO_2_, and more severe nocturnal hypoxia. Additionally, these patients had higher ArI levels and greater sleep fragmentation. However, the mechanisms underlying the interactions between OSA and MASLD remain unclear. Nonetheless, existing literature suggests that CIH plays a pivotal role in the interplay between these conditions. CIH caused by OSA may aggravate MASLD and increase the risk of metabolic dysfunction–associated steatohepatitis in patients with obesity [27]. OSA may exacerbate the severity of MASLD by inducing oxidative stress and inflammatory responses via CIH and sleep deprivation [12]. Furthermore, some studies propose that sleep fragmentation and recurrent arousals in patients with OSA may aggravate MASLD, although the precise pathophysiological mechanisms remain to be fully elucidated [28].

The complex pathophysiological network connecting OSA to MASLD has been increasingly elucidated, with IR serving as the central node [29,30]. Additional mechanisms—including oxidative stress, systemic inflammation, and gut dysbiosis [31]—further amplify this intricate interplay. Individuals with OSA exhibit a significantly elevated risk of developing IR compared to the healthy population [28], and IR is not only a crucial factor in the pathogenesis of MASLD but also intricately linked to its associated complications [17]. CIH has emerged as a key driver of metabolic dysfunction upstream of IR. Disruption of the physiological intrahepatic oxygen gradient—a key spatial determinant of MASLD-related liver injury [32]—is likely amplified by OSA-related CIH. The unique microarchitecture of the liver lobule creates a zonated oxygen tension landscape, with the pericentral region being the most hypoxic and susceptible to lipid accumulation and fibrosis. In this context, hypoxia-inducible factors, particularly HIF-2α, have emerged as critical molecular mediators. Studies using advanced liver-zonation-on-a-chip models have demonstrated that HIF-2α upregulation in hypoxic zones promotes lipid accumulation and cellular injury by activating the Wnt/β-catenin signaling pathway, thereby driving MASLD progression [33]. Collectively, these data provide a plausible mechanistic rationale for our observation that IR surrogates such as TyG-BMI, which reflect metabolic dysfunction potentially exacerbated by hypoxic stress, strongly predict MASLD in patients with OSA.

In recent years, the TyG index has gained widespread application owing to its predictive value in metabolic disorders [34]. As a derivative of the TyG index, TyG-BMI offers a more comprehensive assessment of the body’s metabolic load by incorporating obesity and IR factors [35]. The TG/HDL-C ratio is also considered to be a straightforward and effective surrogate marker of IR because of its strong correlation with IR [36]. Our findings demonstrated that compared to the OSA-only group, the OSA-with-MASLD group exhibited significantly higher TyG-BMI and TG/HDL-C ratios. This finding further substantiates the involvement of IR in the interaction between OSA and MASLD.

TyG-BMI exhibits a significant positive correlation with the prevalence of MASLD, demonstrating notable predictive value [37]. However, there is a paucity of studies on the prediction of MASLD in patients with OSA using TyG-BMI. This study determined that TyG-BMI has substantial predictive value and diagnostic significance. We developed a novel predictive model by combining TyG-BMI with ALT and ALP levels. The model integrating TyG-BMI and ALT yielded an AUC value of 0.921, with an optimal cutoff value of 0.696, sensitivity of 76.7%, and specificity of 92.9%. In contrast, the model combining TyG-BMI and ALP achieved an AUC value of 0.854, an optimal cutoff value of 0.595, a sensitivity of 73.8%, and a specificity of 85.7%. These findings suggest that the predictive model combining TyG-BMI and ALT levels offers the highest AUC value, along with superior predictive value and diagnostic accuracy.

The TG/HDL-C ratio is considered a reliable surrogate marker of IR and exhibits an inverted U-shaped association with risk of MASLD [38]. Integration with additional biomarkers may enhance its clinical utility [39]. Nevertheless, there is a paucity of research examining the predictive capacity of TG/HDL-C ratio for MASLD in patients with OSA. The findings of this study demonstrate that TG/HDL-C ratio is a valuable predictor of MASLD incidence in individuals with OSA. Novel predictive models were developed by combining TG/HDL-C ratio with ALT and ALP levels. The model incorporating TG/HDL-C and ALT exhibited the highest AUC value of 0.920, with an optimal cutoff value of 0.728, sensitivity of 0.835, and specificity of 0.893. Conversely, the optimal cut-off values for the TG/HDL-C and ALP models were 0.562, with a sensitivity of 0.883 and a specificity of 0.679. These results suggest that the TG/HDL-C ratio and ALT level models provide more accurate predictions, higher specificity, and greater diagnostic significance for patients with OSA and concurrent MASLD. The clinical relevance of our predictive models is further supported by a recent translational study demonstrating that 18 months of CPAP therapy in OSA patients not only improved IR and lipid profiles but also significantly reduced MASLD risk scores (Fatty Liver Index and OWLiver test) [40]. This interventional evidence reinforces the causal link between OSA-related hypoxia and metabolic dysfunction and underscores the importance of identifying high-risk OSA patients using simple tools, such as TyG-BMI and TG/HDL-C, as they may derive the greatest benefit from hypoxia correction therapies.

This study provides a systematic evaluation of the predictive performance of TyG-BMI and the TG/HDL-C ratio for MASLD in a well-characterized cohort of patients with polysomnography-confirmed OSA. The integration of comprehensive clinical, laboratory, and polysomnographic data enabled robust multivariable analyses with adjustment for key confounders, including obesity, age, and sleep-related parameters. However, this study has several limitations. First, as a single-center retrospective study with a relatively modest sample size, it is subject to potential selection bias and limited generalizability. Second, MASLD was diagnosed using abdominal ultrasonography rather than liver biopsy, precluding the assessment of inflammatory activity (steatohepatitis) and fibrosis stage. Third, although multivariate analysis was adjusted for major confounders, residual confounding from unmeasured variables (e.g., lifestyle factors and genetic polymorphisms) cannot be excluded. Fourth, the cross-sectional design limits causal inference, and the identified optimal cutoff values require external validation in prospective cohorts. Notably, age emerged as a protective factor against the co-occurrence of OSA and MASLD in multivariate analysis. Despite the overall trend of increasing prevalence of both conditions with advancing age, this seemingly paradoxical finding may be explained by the exclusion of older adults with severe cardiovascular or cerebrovascular diseases and those taking metabolically active medications, who were systematically excluded from our study. This selection bias likely contributes to the observed protective effects of age in our cohort. Future studies leveraging advanced imaging modalities, direct mechanistic biomarkers, and multicenter prospective designs are warranted to confirm these associations, as well as extend our findings.

## 5. Conclusions

In summary, TyG-BMI and TG/HDL-C ratio are effective predictors of MASLD in patients with OSA. Incorporating ALT significantly enhanced predictive accuracy, highlighting the synergistic value of combining metabolic and liver-specific biomarkers. Given their accessibility and low cost, these indices are well-suited for widespread implementation in resource-constrained settings facing the rising dual burden of OSA and MASLD. Future research that integrates these markers with novel indicators may further advance risk prediction and clinical assessment in this population.

## Figures and Tables

**Table 1:** Comparison of baseline characteristics and laboratory data between two groups.

	OSA (*n* = 29)	OSA with MASLD (*n* = 104)	χ^2^/*t*/*Z*	*p*
Age (years)	62.0 (49.5, 66.5)	45.0 (38.0, 53.0)	−3.996	<0.001
Male, n (%)	13 (44.8%)	95 (88.5%)	25.977	<0.001
BMI (kg/m^2^)	24.0 (21.6, 27.0)	29.07(26.69, 32.61)	−5.460	<0.001
AHI (times/h)	14.3 (10.5, 34.9)	53.0 (29.5, 70.3)	−4.850	<0.001
ArI (times/h)	15.0 (11.4, 28.9)	37.4 (20.6, 54.7)	−4.302	<0.001
ODI (times/h)	11.1 (5.5, 24.1)	43.3 (24.9, 66.5)	−5.201	<0.001
MinSpO_2_%	86.0(82.5, 89.0)	79.0 (69.3, 85.8)	−4.292	<0.001
MeanSpO_2_%	95.0 (94.0, 96.0)	94.0 (92.0, 95.0)	−3.922	<0.001
T90%	0.50 (0, 3.05)	6.65 (1.00, 20.63)	−4.039	<0.001
ALT (U/L)	17.0 (11.5, 22.0)	36.0 (24.3, 59.8)	−6.490	<0.001
AST (U/L)	18.0 (15.5, 20.5)	25.0 (19.0, 33.0)	−4.666	<0.001
ALP (U/L)	68.0 ± 14.9	78.3 ± 18.7	2.678	0.008
GGT (U/L)	20.0 (16.5, 31.5)	45.0 (30.0, 76.0)	−5.467	<0.001
FPG (mg/dL)	89.5 (82.8, 93.2)	95.5 (87.5, 103.4)	−2.926	0.003
TC (mmol/L)	4.65 ± 0.84	5.06 ± 0.98	2.087	0.039
TG (mg/dL)	71.8 (65.1, 104.1)	153.7 (104.6, 194.0)	−5.760	<0.001
HDL-C (mmol/L)	1.2 (1.0, 1.6)	1.1 (0.9, 1.2)	−2.668	0.008
LDL-C(mmol/L)	2.5 ± 0.6	3.0 ± 0.7	2.909	0.004
TyG-BMI	195.0 (169.5, 229.7)	252.6 (231.9, 280.5)	−5.422	<0.001
TG/HDLC ratio	0.64 (0.45,1.19)	1.58 (1.11, 2.23)	−5.400	<0.001

**Table 2:** Comparison of clinical data in different groups categorized of TyG-BMI and TG/HDL-C ratio.

	TyG-BMI				TG/HDL-C Ratio			
	<227.80 (A1 Group, *n* = 44)	227.80–260.46 (A2 Group, *n* = 44)	≥260.46 (A3 Group, *n* = 45)	*p*	<1.05 (B1 Group, *n* = 44)	1.05–1.80 (B2 Group, *n* = 43)	≥1.80 (B3 Group, *n* = 46)	*p*
Age (years)	52.5 (42.00, 63.8)	49.5 (41.5, 58.8)	42.0 (36.0, 51.5)	0.003	57.5 (39.8, 63.8)	49.0 (41.0, 57.0)	42.5 (36.8, 53.0)	0.004
Male, n (%)	26.0 (59.1%)	39.0 (88.6%)	40.0 (88.9%)	<0.001	25.0 (56.8%)	37.0 (86.0%)	43.0 (93.5%)	<0.001
BMI (kg/m^2^)	24.5 (22.1, 26.1)	28.4 (26.7, 29.1)	32.8 (30.4, 35.8)	<0.001	25.4 (22.3, 29.7)	28.4 (26.2, 30.1)	30.3 (26.8, 34.8)	<0.001
AHI (times/h)	25.6 (12.6, 55.2)	45.5 (19.4, 63.5)	67.0 (35.7, 78.4)	<0.001	27.7 (11.7, 57.8)	53.5 (23.8, 68.3)	49.1 (30.3, 77.5)	0.002
ArI (times/h)	21.8 (12.8, 41.6)	30.3 (19.5, 47.8)	46.5 (18.8, 62.1)	0.009	22.3 (12.1, 41.5)	33.5 (19.4, 51.4)	38.1 (17.1, 61.5)	0.013
ODI (times/h)	17.8 (6.0, 37.0)	42.7 (17.3, 59.4)	52.1 (30.3, 79.0)	<0.001	21.6 (6.0, 42.7)	45.8 (18.8, 64.8)	41.4 (26.6, 69.3)	0.001
MinSpO_2_%	85.5 (80.0, 89.8)	81.0 (73.5, 86.0)	76.0 (65.0, 80.5)	<0.001	84.0 (77.5, 88.0)	80.0 (70.0, 85.0)	79.5 (70.5, 86.0)	0.016
MeanSpO_2_%	95.0 (94.3, 96.0)	94.0 (93.0, 95.0)	93.0 (89.5, 95.0)	<0.001	95.0 (93.3, 96.0)	94.0 (93.0, 95.0)	94.0 (92.0, 95.0)	0.085
T90%	0.50 (0.00, 3.18)	6.40 (0.95,13.40)	16.80 (1.95, 41.45)	<0.001	1.00 (0.00,6.95)	6.50 (1.00,20.10)	7.30 (0.78, 22.48)	0.006
ALT (U/L)	20.0 (15.3, 28.0)	29.0 (22.3, 45.5)	45.0 (29.0, 68.0)	<0.001	22.0 (13.5, 35.0)	29.0 (22.0, 47.0)	36.0 (27.8, 63.0)	<0.001
AST (U/L)	19.0 (16.0, 26.8)	21.0 (18.0, 27.8)	26.0 (20.0, 38.0)	0.001	20.0 (17.3, 25.5)	21.0 (18.0, 29.0)	26.0 (20.0, 36.3)	0.015
ALP (U/L)	73.0 (65.0, 80.0)	76.0 (64.0, 84.0)	76.0 (65.5, 94.0)	0.468	70.0 (65.0, 81.0)	74.5 (64.0, 83.3)	79.5 (68.0, 94.5)	0.057
GGT (U/L)	26.5 (17.3, 54.5)	34.0 (26.0, 63.0)	48.0 (37.0, 88.0)	<0.001	24.0 (17.0, 46.0)	34.5 (27.5, 68.3)	50.5 (36.3, 102.5)	<0.001
FPG (mg/dL)	90.7 (83.3, 97.4)	93.4 (86.3, 103.0)	95.6 (87.7, 104.4)	0.144	91.3 (83.8, 97.4)	94.3 (86.2, 107.6)	95.6 (87.4, 101.7)	0.210
TC (mmol/L)	5.0 ± 1.2	4.8 ± 0.9	5.1 ± 0.7	0.141	4.88 (4.35, 5.64)	4.9 (4.1, 5.6)	5.0 (4.6, 5.8)	0.269
TG (mg/dL)	83.3 (65.8, 149.3)	125.4 (103.0, 161.0)	176.3 (132.9, 264.0)	<0.001	75.3 (64.9, 95.9)	128.5 (103.7, 153.3)	203.3 (176.3, 292.8)	<0.001
HDL-C (mmol/L)	1.3 ± 0.3	1.1 ± 0.2	1.0 ± 0.2	<0.001	1.3 (1.1, 1.6)	1.0 (0.9, 1.2)	0.9 (0.9, 1.1)	<0.001
LDL-C (mmol/L)	2.8 (2.2, 3.3)	2.9 (2.3, 3.2)	3.0 (2.6, 3.4)	0.264	2.7 ± 0.7	2.9 ± 0.8	3.1 ± 0.7	0.026

**Table 3:** Predictive performance of TyG-BMI and TG/HDL-C Ratio, alone and in combination with ALT or ALP, for MASLD in OSA.

		*p*	AUC	Sensitivity (%)	Specificity (%)	Youden Index
TyG-BMI	Alone	<0.001	0.826	79.60	75.00	0.546
	Combined with ALT	<0.001	0.921	76.70	92.90	0.696
	Combined with ALP	<0.001	0.854	73.80	85.70	0.595
TG/HDL-C ratio	Alone	<0.001	0.824	93.20%	60.70%	0.539
	Combined with ALT	<0.001	0.920	83.50%	89.30%	0.728
	Combined with ALP	<0.001	0.840	88.30%	67.90%	0.562

## Data Availability

Reasonable requests to access related data will be considered on a case-by-case basis and should be made to the corresponding author (Jingwei Mao, maojingwei@dmu.edu.cn). Ethical approval for data-sharing agreements is required to protect participants’ confidentiality.

## Supplementary Materials

The following supporting information can be downloaded at: https://www.mdpi.com/article/10.3390/jcm15051859/s1, Table S1: Collinearity analysis of relevant variables.

## Abbreviations

The following abbreviations are used in this manuscript:

TyG-BMI	Triglyceride-glucose index with body mass index
TG/HDL-C	Triglyceride-to-high-density-lipoprotein-cholesterol
MASLD	Metabolic dysfunction-associated steatotic liver disease
NAFLD	Nonalcoholic Fatty Liver Disease
OSA	Obstructive sleep apnea
ALT	Alanine aminotransferase
CIH	Chronic intermittent hypoxemia
IR	Insulin resistance
FPG	Fasting plasma glucose
LDL-C	Low-density lipoprotein cholesterol
AST	Aspartate aminotransferase
ALP	Alkaline phosphatase
GGT	Gamma-glutamyltransferase
AHI	Apnea–hypopnea index
ArI	Arousal index
ODI	Oxygen desaturation index
ORs	Odds ratios
CIs	Confidence intervals
AUC	Areas under the curve
TyG index	(ln[TG(mg/dL) × FPG(mg/dL)]/2)
TyG-BMI	TyG × BMI
TG/HDL-C ratio	TG (mmol/L) divided by HDL-C (mmol/L)
