# Supplementary material for: The Predictive Value of TyG-BMI and TG/HDL-C for Metabolic Dysfunction-Associated Steatotic Liver Disease in Obstructive Sleep Apnea: A Single-Center Retrospective Cohort Analysis
Source: J Clin Med. 2026 Feb 28;15(5):1859. doi: 10.3390/jcm15051859 (PMC12985736; doi:10.3390/jcm15051859)
Supplement: Supplementary file 1 [file jcm-15-01859-s001.zip › jcm-4148287-supplementary.pdf]

Table S1. Collinearity analysis of relevant variables

Variable	Collinearity Statistics	
	Tolerance	VIF
Age	0.687	1.455
Sex	0.717	1.394
AHI	0.073	13.678
$MinSpO_2$	0.265	3.778
$MeanSpO_2$	0.211	4.741
T90%	0.181	5.539
ArI	0.179	5.585
ODI	0.077	13.021
ALT	0.168	5.961
AST	0.185	5.393
ALP	0.805	1.242
GGT	0.488	2.049
LDL-C	0.895	1.118
TyG-BMI	0.535	1.869
TG/HDLC ratio	0.579	1.726

Note: AHI, apnea-hypopnea index; ArI arousal index; ODI, oxygen desaturation index; ALT, alanine aminotransferase; AST, aspartate aminotransferase; ALP, alkaline phosphatase; GGT, gamma-glutamyltransferase; LDL-C, low-density lipoprotein cholesterol; TG, triglycerides; HDL-C, high-density lipoprotein cholesterol.
